# Effects of Housing System on Anxiety, Chronic Stress, Fear, and Immune Function in Bovan Brown Laying Hens

**DOI:** 10.3390/ani12141803

**Published:** 2022-07-14

**Authors:** Andrew M. Campbell, Alexa M. Johnson, Michael E. Persia, Leonie Jacobs

**Affiliations:** School of Animal Sciences, Virginia Tech, Blacksburg, VA 24061, USA; drewc1@vt.edu (A.M.C.); alexaj3@vt.edu (A.M.J.); mpersia@vt.edu (M.E.P.)

**Keywords:** attention bias, conventional cage, environmental complexity, enrichment, feather corticosterone, IgA, laying hen

## Abstract

**Simple Summary:**

The objectives of this study were to determine if housing Bovan brown laying hens in conventional cages or enriched floor pens impacted novel physiological and behavioral markers for animal welfare and whether we can use these markers to assess animal welfare. We found that birds that were housed in conventional cages showed increased tonic immobility durations (indication of fearfulness), decreased fecal Immunoglobulin A (indicator of immune function), and increased feather corticosterone concentrations (indicator of chronic stress) compared to hens that were housed in enriched pens. These results indicate that caged birds are more stressed, have reduced immune function, and are more fearful than birds that are housed in pens. In contrast to expectations, we found that caged hens showed a shorter latency to feed during attention bias testing, indicating reduced anxiety compared to birds from pens. Overall, we found that conventional cages generally impacted animal welfare negatively, with the exception of anxiety. In addition, the results suggest that the chosen novel markers for animal welfare show appropriate contrast between long-term housing systems for laying hens. Yet, additional work needs to be done before these measures can be used more broadly.

**Abstract:**

The scientific community needs objective measures to appropriately assess animal welfare. The study objective was to assess the impact of housing system on novel physiological and behavioral measurements of animal welfare for laying hens, including secretory and plasma Immunoglobulin (IgA; immune function), feather corticosterone (chronic stress), and attention bias testing (ABT; anxiety), in addition to the well-validated tonic immobility test (TI; fearfulness). To test this, 184 Bovan brown hens were housed in 28 conventional cages (3 birds/cage) and 4 enriched pens (25 birds/pen). Feces, blood, and feathers were collected 4 times between week 22 and 43 to quantify secretory and plasma IgA and feather corticosterone concentrations. TI tests and ABT were performed once. Hens that were from cages tended to show longer TI, had increased feather corticosterone, and decreased secretory IgA at 22 weeks of age. The caged hens fed quicker, and more hens fed during the ABT compared to the penned hens. Hens that were in conventional cages showed somewhat poorer welfare outcomes than the hens in enriched pens, as indicated by increased chronic stress, decreased immune function at 22 weeks of age but no other ages, somewhat increased fear, but reduced anxiety. Overall, these novel markers show some appropriate contrast between housing treatments and may be useful in an animal welfare assessment context for laying hens. More research is needed to confirm these findings.

## 1. Introduction

Animal welfare is a multifaceted concept that involves an animal’s ability to interact and cope with its environment. Good animal welfare would be achieved when an animal is allowed to display natural behaviors (natural living), be healthy and function normally (basic health and functioning), and experience a generally positive emotional state (affective state) [1]. For laying hens, commercial housing systems such as conventional cages can negatively impact animal welfare by restricting natural behaviors, causing health and functioning concerns such as cage layer fatigue, and likely results in worse affective states (anxiety and fear) [2]. While aspects of natural living and basic health and functioning are relatively easily measured and well-studied, few studies investigate the effects of housing system on the affective states of laying hens. Additionally, as nearly all measures of affective state rely on interpretations of animal behavior, there is need for additional physiological measurements to allow for a more cumulative assessment of animal affective states. 

With limited physiological measures of emotion and affective state available, most insight comes from behavioral assessments of negative emotions such as fear. Fear is a short-term emotional response to a current threat and elicits either a freeze, fight, or flight response [3,4,5]. A common assessment of animal emotion is performed using fear tests, such as a novel object test, human approach test, or tonic immobility (TI) test [6]. Measuring fear using TI is well-documented in poultry and utilizes their natural prey-predator behavioral response. TI is a type of freezing behavior where birds will feign death and is used when captured by a predator [7]. Previous investigations of TI in relation to housing systems had variable results. For example, barren caging conditions have resulted in longer TI durations, thus greater levels of fear, in Hyline Brown laying hens when compared to more enriched conditions [8,9]. However, other studies have found no effect of housing system on TI duration in laying hens [10,11]. These variable results highlight the need for further investigations of TI and other measures to elucidate the effects of housing system on emotion and affect. 

Affective state is a long-term mood state which comes from the culmination of experiences and emotional responses. An animal’s life experiences elicit short-term emotional responses [12]. These responses culminate to form a mood, which can range from positive to negative in valence, and shapes the animal’s affective state. Affective states influence how animals make decisions and can bias their cognitive reasoning, which can then be used to infer the affective state based on behaviors that indicate information-processing [12,13,14]. One cognitive bias test that was previously applied in chickens is the attention bias test (ABT). The ABT is a validated and well-used method to measure affective states, more specifically anxiety, in agricultural animals [13]. In an ABT, the level of vigilance or attention an animal allocates to a perceived threat is quantified [13]. This allocation of vigilance is differential and affect-mediated, with more anxious affective states resulting in more vigilance towards a threatening stimulus [3,13]. The ABT was validated in laying hens, with birds that were given anxiogenic drugs displaying increased vigilance compared to control birds [15]. Excess anxiety decreases the ability of commercial poultry to cope with changes in their environment such as transport, handling, and loud noises. In laying hens, the ABT reflected anxiety that was related to range usage [15,16]. Determining anxiety via the ABT was also successful in broilers [3], sheep [17,18], and pigs [19]. However, ABT has not yet been applied to assess the impact of housing system (cage vs. cage-free) environments on laying hen anxiety. 

Feather corticosterone (CORT) is a potential promising physiological biomarker for chronic stress [20]. Concentrations of feather CORT can provide a retrospective view on stressful experiences during feather growth [21]. The calamus of a feather is highly vascularized which allows for the deposition of circulating CORT into the feather as it grows, allowing for quantification of hypothalamic-pituitary-adrenal (HPA) axis activity over a prolonged period of time [20]. The extraction and quantification of feather CORT has been validated for use in layers, broilers, turkeys, and non-domesticated bird species and requires no invasive sampling procedures [20,21,22,23]. Feather CORT could provide insight in chronic stress that is caused by housing systems in laying hens. 

Secretory Immunoglobulin-A (IgA) is the most abundant antibody on mucosal surfaces including the intestinal tract and has an important role in mediating the adaptive humoral immune defense [24,25,26]. In addition, IgA circulates in the blood, yet the role is less understood and likely serves to help reduce inflammation [26]. Concentrations of IgA also seem to reflect the valence (positivity or negativity) of environmental stimuli [18]. For example, IgA concentrations are downregulated in response to physical or psychological stress [27,28,29,30]. Broiler chickens and laying hens under prolonged periods of heat stress (chronic negative stressor) showed decreased concentrations of plasma IgA when compared to the control treatment [31,32]. Mice that were exposed to restraint stress over four days (negative stressor) showed decreased concentrations of intestinal IgA compared to the control [29]. Shelter cats with access to enrichment (positive stimuli) had higher levels of secretory (fecal) IgA than cats without access to enrichments [33]. In mice, prolonged voluntary exercise (a high arousal-positive-valence activity) increased salivary IgA concentrations after 3 weeks, indicating that IgA concentrations can increase in response to positive activities [34]. In addition, forced prolonged exercise (a high arousal-negative-valence activity) had the opposite effect in horses, rats, and humans [26,35,36,37]. The valence-dependent response of circulating and secretory IgA indicates the potential for use as a marker for the affective state. 

The combined use of novel and well-validated measures for emotion and affective state could provide a better understanding of the impacts of different housing conditions on laying hen welfare. In addition, it allows further confirmation of novel measures as we can compare novel test outcomes with well-validated test responses, such as the TI test. Therefore, the objectives of this study are to (1) determine if the housing system impacts laying hen welfare outcomes that are related to the affective state and emotion and (2) determine if these novel measures can be used to assess laying hen welfare. We hypothesized that birds that were housed in enriched floor pens (pen) would show decreased fear and anxiety, increased IgA concentrations, and decreased feather CORT concentrations than birds that were housed in traditional conventional caging, indicating that these novel measures can be used to assess laying hen welfare and that enriched housing systems contribute to positive affective states in pen laying hens. 

## 2. Materials and Methods

### 2.1. Birds and Housing Treatments

This experiment was approved by the Virginia Tech Institutional Animal Care and Use Committee (IACUC protocol 18-205). Day-old Bovan Brown chicks (*n* = 184) were sourced from a commercial hatchery (Blackstone, VA, USA) where they were beak trimmed after hatch. The birds received a Salmonella vaccination at 16 weeks of age. From day one to week six of age, 84 chicks were reared in conventional cages and 100 chicks in floor pens as part of an unrelated study that was investigating the impact of dietary phosphorous on egg production. 

In week six, all the birds were wing banded for individual identification, relocated to another facility, and regrouped, yet housing treatments (either cage or floor pen) remained consistent. The chicks from floor pens were randomly distributed over four enriched floor pens (pen of 16.7 m^2^), with 25 birds per pen. The chicks from cages were distributed over 14 conventional cages (cage) of 0.093 m^2^, with six birds per cage until week 12 of age. At week 12, three birds from each cage were moved to 14 unoccupied cages to reduce the stocking density, resulting in 28 cages total with three birds per cage. The penned birds were not regrouped at 12 weeks of age. All the pens contained pine wood shavings (7 cm depth), one trough feeder (92.7 cm^2^/bird) and bell drinker (Plasson, Ma’agan Michael, Israel), 10 galvanized steel nest boxes that were arranged in two tiers of five boxes (30.5 cm long × 30.5 cm wide × 35.6 cm high), one hay bale (replaced as needed), and a head of cabbage that was provided twice per week, which was suspended from the ceiling at bird level. The birds had access to perches (5.49 m or 0.22 m/bird perch space) consisting of pressure-treated 5 cm × 10 cm boards for the frame and three 2.5 cm diameter PVC pipes that were mounted at 45 cm, 60 cm, and 90 cm heights.

There were four rows of stacked conventional cages (38 cm wide × 38 cm long × 46 cm high) with a sloped wire floor that were located in the same room as the pens, and contained two nipple drinkers per cage, a feed trough that was suspended outside the cage with a gap in the wire for birds to access feed, and an egg collection trough on the opposite side. The pen space allowance was 0.668 m^2^/bird, compared to 0.031 m^2^/bird in the cage housing after week 12. The birds had *ad libitum* access to feed and water. From hatch to week six, the pullets were fed an experimental diet as part of the phosphorous experiment. Following week six, the birds were phase fed diets that were formulated to meet their nutritional needs that were appropriate for their age and developmental status. Lighting included 12 h light and 12 h dark, with windows allowing for natural light exposure during the daytime. Daylight exposure was equal between the treatments. Temperatures within the house were managed by assessing bird comfort based on behavioral responses (huddling when cold/panting when warm), however, a cold period in the winter reduced the in-house temperatures to a minimum of 5 °C when the birds were 33–37 weeks of age. 

### 2.2. Behavioral Measurements

A TI test was performed to assess fearfulness on six arbitrarily selected hens per pen (total of 24 birds) and on one hen per cage from 24 randomly selected cages (total of 24 hens) at 23 weeks of age. The test was performed by a single researcher in the hallway of the room in which the hens were housed as described in [3]. The hens were placed on their backs in a V-shaped wooden cradle and restrained by the researcher by placing one hand on the sternum and cupping the head with the other hand. After 15 s of restraint, the researcher stepped away without making eye contact. Following the induction of TI, the duration (s) was recorded to determine fearfulness. If the induction of TI was unsuccessful, the researcher attempted to induce TI again for a maximum of three times. If TI was not induced in three attempts, the latency to rightening was scored as 0 s. The maximum duration of TI was 300 s. 

An attention bias test (ABT) was performed by two observers to assess anxiety using a modified method as described in [3] on nine hens per pen and three hens per cage for 12 arbitrarily selected cages at 30 weeks of age. One bird from each cage that was tested during ABT was also tested for TI. The inter-observer agreement was tested for latency to start feeding of 12 hens and was good among the two observers (Cronbach’s α of 0.841). The test arena consisted of plastic paneling and rubber flooring (76.2 cm × 76.2 cm), and contained a trough feeder with feed, mealworms, and oats. The arena was located in a separate room near the hens’ room but far enough to block out the alarm call in the hens’ room. The birds were tested in familiar groups of three hens. After placement of three hens in the arena, a conspecific ground predator alarm call was played for 8 s. Following the alarm call, the number of birds and latencies to begin feeding (s) were recorded. Video recordings (EOS Rebel T7 DSLR Camera, Canon, Tokyo, Japan) were used to determine the occurrence (yes/no) of vigilant behaviors (freeze, neck stretches, looking around, and erect posture) within the first 30 s of testing. Each of the four vigilance behaviors were scored as a 1 (yes) or 0 (no) and combined to obtain a vigilance score from 0 to 4 [3,16]. The alarm call was replayed for 8 s if one of four scenarios occurred (Table 1). Birds that did not start feeding after the first alarm call received a maximum latency of 300 s. After the second alarm call, the number of birds that were feeding and the latency to resume feeding (s) were recorded.

### 2.3. Molecular Measurements

#### Feces and Blood

A total of 4 fecal samples and 12 plasma samples per treatment per time point were collected during weeks 22, 25, 29, and week 43 of age to determine the fecal and plasma IgA concentrations. Fresh fecal samples were collected from the cage or pen floors and pooled in microcentrifuge tubes. The birds were arbitrarily selected from floor pens (*n* = 3/pen) or cages (*n* = 1 bird/cage) for blood sampling during each time point. Across ages, nine birds were selected twice, and 30 birds were selected once. 

For fecal samples, visual inspection and observation of defecation were used to ensure the freshness of the samples and to prevent degradation of fecal IgA by fecal proteases. Following collection, the fecal samples were placed on ice and then stored at −80 °C. Fecal IgA was quantified using the total protein extraction with a saline extraction method that was similar to that described in [38,39,40]. A total of 10 mL of a saline extraction buffer (0.01 M phosphate-buffered saline, 0.5% Tween (Sigma-Aldrich, St. Louis, MO, USA), and 0.05% sodium azide) was added to each 1 g fecal sample, followed by homogenization. Fecal suspensions were centrifuged at 1500× *g* for 20 min at 5 °C and the supernatant was removed and placed in microcentrifuge tubes. Then, 20 µL of protease inhibitor cocktail (Sigma-Aldrich, St. Louis, MO, USA) was added to the supernatant and homogenized before storage at −20 °C until analysis.

Blood (1 mL) was drawn from the brachial vein and collected in glass tubes containing 0.05% EDTA for anticoagulation. The sample collection times (s) were recorded from start of handling until the removal of the needle, to ensure that all the samplings occurred in under two minutes to minimize the potential effects of sampling stress. Prolonged handling can impact acute-stress-related blood parameters [41], although it is unknown whether this is the case for IgA. The mean sample collection time (±SD) was 77 ± 30 s and IgA concentrations showed no correlation with collection times (R^2^ = 0.0007; *p* = 0.80). The sample vials were then lightly mixed via inversion before storage on ice. Then, the blood samples were centrifuged at 10,000× *g* for 10 min at room temperature, after which the plasma was removed and aliquoted into sterile microcentrifuge tubes and stored at −20 °C until analysis. The plasma and fecal samples were analyzed for IgA concentrations [µg/µL] via a commercial ELISA kit (Abcam, Cambridge, MA, USA) following the manufacturer instructions. The intra-assay CV% were below 2% for all the samples (min: 0.005; max: 1.1%).

### 2.4. Feathers 

Tail feathers (*n* = 6/treatment per timepoint; 48 samples total) were collected during weeks 22, 25, 29, and 43 of age to determine the feather CORT concentrations. The tail feather samples were collected by cutting the calamus as close to the skin as possible without contacting or damaging the skin. At each sampling timepoint, different tail feathers were collected, thus the same tail feather was never collected more than once. The birds were arbitrarily selected from floor pens (3 birds/pen) and conventional cages (1 bird/cage) for feather sampling at each timepoint. Across ages, six birds were selected twice, and 36 birds were selected once. Following collection, the feathers were stored in Whirl Pac bags (Nasco, Fort Atkinson, WI, USA) and stored at −20 °C until assay. Visual inspection ensured that the least damaged feathers were selected for the assay. The feather CORT concentrations were determined following an extraction procedure that was described in [20]. First, the feathers were weighed (mg) to standardize the feather CORT concentrations by feather weight. The feathers were finely minced (including vane and rachis) using surgical scissors (<5 mm sections) into 20 mL scintillation vials. Following mincing, 1 mL of methanol was added and the vials were placed in a sonicating water bath at room temperature for 30 min. The samples were then placed in a shaking hot water bath (Jouan Inc., Precision Sci. Div. Chicago, IL, USA) at 56 °C overnight. The samples were filtered to remove feather material and the filtrate was transferred to scintillation vials. The methanol was allowed to evaporate completely under a fume hood and CORT was reconstituted in 1 mL ELISA buffer. Reconstituted CORT concentrations were assayed using a commercial ELISA kit (Abcam, Cambridge, MA, USA) following the manufacturers protocol. The intra-assay CV% were below 13% for all the feather CORT samples (range: 0.15–12.50%).

### 2.5. Statistical Analysis

All statistical analyses were performed in JMP Pro 15 (SAS institute, Cary, NC, USA). Pen or cage was considered the experimental unit for all the response variables. Bird was considered the observational unit for all response variables besides fecal IgA where pen or cage was the observational unit due to pooling of the samples. The distribution of residuals of all the dependent variables were visually inspected using a normal quantile plot to determine normalcy. Normally distributed dependent variables included TI duration (s), ABT latency to begin feeding (s), latency to resume feeding (s), plasma and fecal IgA concentrations (ng/mL), and feather CORT concentrations (ng/mg). Normally distributed data were analyzed using general linear mixed models. Fixed effects were housing system (pen or cage), age (weeks 22, 25, 29, 43) and their interaction. For all the response variables besides fecal IgA, mixed models were used with pen or cage number and bird ID as random effects so that the model identifies the unit (cage or pen) to which the treatment was randomly assigned and independently applied. Non-significant interactions (*p* > 0.1) were removed from the model. Post-hoc analysis was done using Tukey HSD testing. Dependent variables without normally distributed residuals were tested using a nonparametric Wilcoxon Rank sum test. These variables included ABT birds (%) which began and resumed feeding, total vigilance scores (1–4), and birds (%) displaying vigilance behaviors. Associations were deemed significant at *p* ≤ 0.05 and trends at *p* ≤ 0.1. The data are presented as LS means ± SEM unless otherwise noted. 

## 3. Results

### 3.1. Behavioral Measures

**Figure 1:**
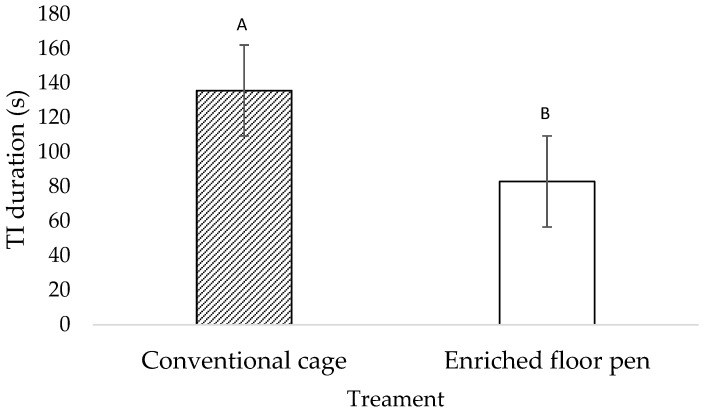
Least square mean estimates (±SEM) of tonic immobility (TI) duration at week 23 of age (*n* = 24 birds/treatment) for hens that were housed in conventional cages and hens that were housed in enriched floor pens. Bars lacking a common superscript tend to differ at *p* < 0.1.

TI duration (Figure 1) tended to be shorter for the pen hens (82.88 ± 35.86 s) compared to the caged hens (135.70 ± 21.78 s; F_1,46_ = 3.16; *p* = 0.080). During ABT, 92.1% of birds (35/38) showed ≥1 vigilance behaviors. After the first alarm call, more cage hens tended to begin feeding compared to the pen birds (χ^2^ = 3.55; *p* = 0.058; Table 2). Latencies to begin feeding were shorter in the cage hens compared to the pen hens (F_1,70_ = −2.33; *p* = 0.022; Table 2). Following the second alarm call, more cage birds resumed feeding compared to the pen birds (χ^2^ = 5.28; *p* = 0.020; Table 2). The latency to resume feeding did not differ between the cage and pen hens (F_1,50_ = 1.20; *p* = 0.279; Table 2). The total vigilance behavior scores (χ^2^ = 0.01; *p* = 0.967) and the frequency of observed vigilance behaviors (all *p* > 0.200) did not differ between the treatments (Table 2). 

### 3.2. Molecular Measures

**Figure 2:**
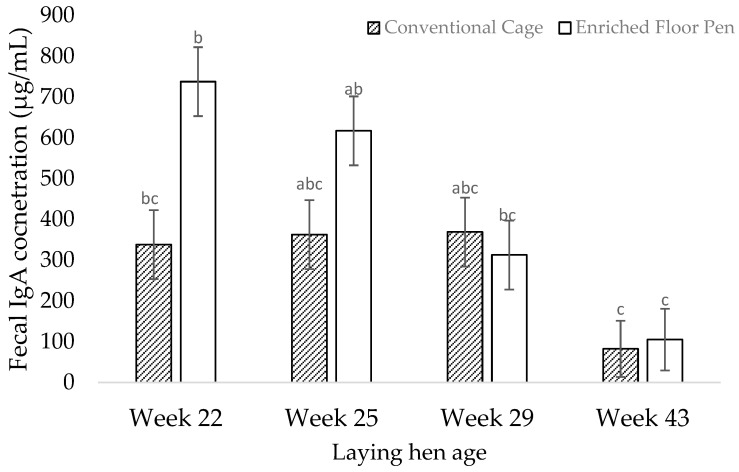
Least square mean estimates (±SEM) of fecal (secretory) immunoglobulin-A (IgA) concentrations in hens that were housed in conventional cages or enriched floor pens during weeks 22, 25, 29, and 43 of age. Bars lacking a common superscript differ at *p* < 0.05.

There was a treatment by age interaction effect on the fecal IgA concentrations (F_1,27_ = 3.51; *p* = 0.035; Figure 2). The fecal IgA concentrations were lower in the cage hens in week 22 (F_1,27_ = −3.38; *p* = 0.049) compared to the pen hens in that week (Figure 2). The fecal IgA concentrations did not differ between the treatments in weeks 25 (F_1,27_ = −1.94; *p* = 0.540), 29 (F_1,27_ = 0.23; *p* = 0.999), and 43 (F_1,27_ = −0.37; *p* = 0.999). The fecal IgA concentrations in the pen layers were higher in weeks 22 compared to week 29 (F_1,27_ = 4.23; *p* = 0.008) and week 43 (F_1,27_ = 6.06; *p* = 0.002). The fecal IgA concentrations were higher in the pen hens in week 25 when compared to week 43 (F_1,27_ = 4.85; *p* = 0.002). No difference in the fecal IgA concentrations were found in the cage hens over time (*p* > 0.1). 

Plasma IgA concentrations were not impacted by treatment (F_1,88_ = 0.66; *p* = 0.419) or treatment by week interaction (F_1,48_ = −1.63; *p* = 0.110). The plasma IgA concentrations were lower in week 22 (87.25 ± 15.65 µg/mL) compared to week 29 (260.90 ± 15.65 µg/mL; F_1,88_ = −7.69; *p* < 0.001) and 43 (201.80 ± 14.45 µg/mL; F_1,88_ = −5.36; *p* < 0.001), and lower in week 25 (104.55 ± 16.45 µg/mL) compared to week 29 (F_1,88_ = −7.00; *p* < 0.001) and week 43 (F_1,88_ = −4.44; *p* < 0.001). The plasma IgA concentrations in week 29 were higher than in week 43 (F_1,88_ = 2.82; *p* = 0.030). 

**Figure 3:**
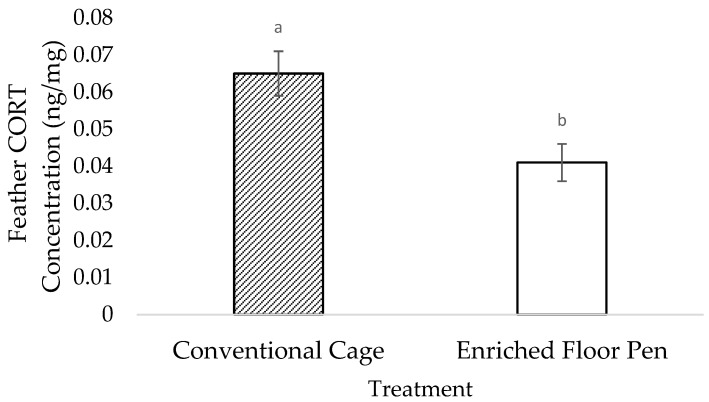
Least square means estimates (±SEM) of the total feather corticosterone (CORT) concentrations for laying hens that were housed in conventional cages or in enriched floor pens. *n* = 12 samples/timepoint (6 samples/treatment) from weeks 22, 25, 29, and 43 of age. Bars lacking a common superscript differ at *p* < 0.05.

**Figure 4:**
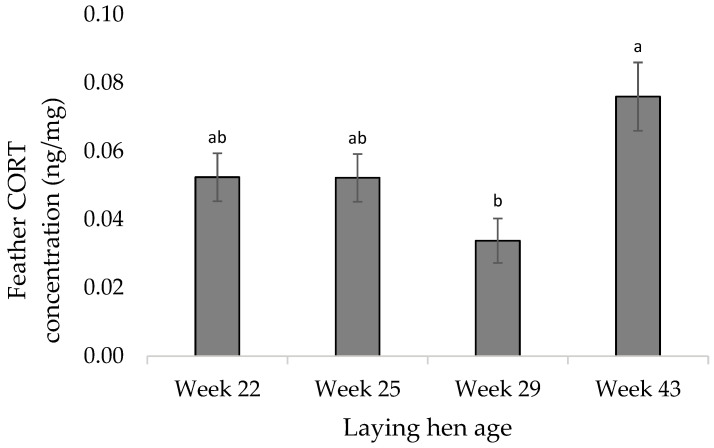
Least square means estimates (±SEM) of the total feather corticosterone (CORT) concentrations by age in weeks. Bars lacking a common superscript differ at *p* < 0.05.

The feather CORT concentrations by feather weight (ng/mg) were higher in the cage hens compared to the pen hens (F_1,43_ = 2.18; *p* = 0.004; Figure 3). The feather CORT concentrations were higher in week 22 and 25 compared to week 29 (F_1,43_ = 6.07; *p* = 0.015; Figure 4), but lower in week 29 compared to week 43 (F_1,20_ = −3.30; *p* = 0.012; Figure 4). There was no treatment by week interaction effect on the feather CORT (F_1,37_ = 1.43; *p* = 0.251).

## 4. Discussion

This study investigated the effects of housing system (enriched floor pens vs. conventional caging) on behavioral and molecular markers for laying hen welfare including TI, attention bias, fecal and plasma IgA concentrations, and feather CORT concentrations. Birds in conventional cages tended to be more fearful based on TI responses, were less anxious based on ABT responses, and experienced more chronic stress based on low IgA levels and high feather CORT levels. Overall, these results suggest that the cage layers experienced a decreased welfare status when compared to pen layers, except in terms of anxiety. This is the first study to show a relationship between housing conditions and IgA and feather CORT responses in laying hens.

### 4.1. Behavioral Measures

Cage housing tended to result in more fearful but less anxious birds compared to pen housing, which is in line with earlier findings [42,43]. Fearfulness is considered a negative emotion and frequent bouts of fear can be indicative of negative affective states in animals [3,44,45]. Similar to our results, cage housing increased the TI duration in 18-week-old (cages: 519 s vs. pens: 471 s) and 1-year-old (cages: 443 s; pens: 189 s) white leghorn hens when compared to floor pen housing [44]. However, these reported durations were longer than those in the current study in Bovan brown laying hens (cages: 136 s vs. pens: 88 s), potentially due to strain differences [46,47]. Although the difference in the current study was only a tendency, we suspect this is due to limited statistical power because of the relatively small sample size, as a large numerical difference in TI durations was observed. Previous studies have found no difference in fear levels related to housing system in commercial laying hens [11,48]. In ideal situations, fear-associated behavior is adaptive and aids in helping animals prevent injury during threatening encounters. However, fear-associated behavior in production housing systems is generally maladaptive, with animals having no ability to escape or appropriately respond to fearful stimuli [49]. Over time, this excessive fear response could lead to frustration, learned helplessness, and eventually negative affective states which can impact productivity. Increased fear can lead to decreased egg production [50,51], increased feather pecking [52,53,54,55], and increased injury rate [56]. In systems that have high fear levels, these impacts could erase, or reverse production gains that are generally attributed to intensive housing systems. The increased level of fear in cages compared to pens could have contributed to a negative affective state in hens in cages.

Cage housing conditions reduced anxiety in laying hens compared to pen housing conditions. Attention bias testing was validated for use in laying hens [15,16], and has been applied to test the effect of environmental conditions on anxiety in starlings [57] and broilers [3]. During the test, 92.1% of birds showed some form of vigilance behavior, indicating that the ABT was sufficiently threatening to achieve anxiety. Contrary to expectations, cage housing decreased the latency to begin feeding and resulted in a higher percentage of hens feeding after the first and second alarm call compared to pen housing. These results indicate that hens that were housed in cages did not bias their attention towards the threat, thus were less anxious than hens that were housed in pens, which is somewhat contradictory to earlier studies [3,15]. Broilers from high-complexity pens were quicker to begin feeding following an alarm call compared to broilers from low-complexity pens (high complexity: 160 s vs. low complexity 214 s; [3]). Outside-ranging laying hens showed shorter latencies to feed compared to hens that never went outside (outdoor: 86 s vs. indoor: 170 s; [16]). Although the treatment conditions were different in both studies compared to the current study, they both involve a level of environmental complexity. In the current study, as in Anderson et al. 2021 [3], the test was performed in groups of three birds, rather than testing birds individually (as in [15]). The cage hens were tested with their cage conspecifics, and pen hens with 3 out of 25 pen conspecifics. It is possible that pen hens experienced this temporary regrouping as more negative than the cage hens, depending on the social hierarchy within the pen. Further testing is needed to confirm the effect individual versus group testing of anxiety, and of these housing conditions on anxiety in laying hens.

Our results support earlier findings that fear and anxiety can be opposing [5,58] although some studies show they can be positively associated [59,60]. Fear is a generally fast adaptive state of vigilance to a present, negatively valanced stimulus which activates a defensive response such as fight, flight, or freeze [4]. Anxiety elicits vigilance and apprehension to non-existing or ambiguous threats [4]. Although the symptoms of fear and anxiety are similar [4], evidence suggests that they are distinct emotional experiences. Research in rodents shows that three stages of defense exist, which include the pre-encounter defense (apprehension to a place where a predator has been seen), a circa-strike defense (physical contact with a predator), and a post-encounter defense (predator is identified at a distance) [4,61,62,63]. Anxiety has been associated with the pre- and post-encounter stages, but not with the circa-strike phase [4]. As the TI test is performed, the researcher acts as a predator coming in physical contact with the hen, inducing a catatonic state. If similar stages of defense exist in chickens, the TI test most likely simulates the circa-strike defense phase. This suggests that the emotional experiences that are tested in the ABT and the TI test are associated with distinct phases of defense. Cage birds may be more fearful in the circa-strike phase and less anxious in the pre- and post-encounter stages compared to the pen birds. Thus, this could explain why the outcomes are opposing. Although these defense phases have not been confirmed in chickens, it is possible that a similar distinction exists. Therefore, it is possible that housing systems impact hen fear and anxiety differently. Overall, the hens’ behavioral responses indicate that anxiety and fear are affected by housing system, with pens tending to reduce fear but not anxiety compared to cages. 

### 4.2. Molecular Measures

The housing treatments impacted fecal IgA concentrations during week 22, resulting in decreased concentrations in the cage layers compared to the pen layers. Our results indicate that birds that are housed in complex, low density environments (enriched floor pens) are under less chronic stress (reflected by immune status) than birds that are housed in barren, highly confined environments (conventional cages) at 22 weeks of age. Additionally, our results show that fecal IgA could be a potential physiological indicator of animal welfare status. This is in line with past findings in swine, rodents, laying hens, and broilers [26,31,32,40,64]. Prolonged heat stress decreased plasma IgA concentrations in broilers and layers [31,32], which showed plasma IgA concentrations in similar ranges as in the current study (previous study: 0.162–0.290 mg/mL vs. current study: 0.087–0.260 mg/mL). 

Secretory and plasma IgA concentrations are promising valence-dependent indicators of stress in certain mammals [28,29,40]. We did not find an impact of housing conditions on plasma IgA concentrations, showing at least in similar contexts, this measure could be less relevant for laying hen welfare assessments. However, to our knowledge this study is the first to investigate secretory and plasma IgA in an animal welfare context in laying hens. Access to litter, containing microorganisms and fecal material, could have impacted the higher secretory IgA concentrations in pens compared to the cage hens at 22 weeks of age. Alternative housing systems (aviary, pens) result in higher concentrations of bacteria and fungi in the air than conventional cage housing [65,66]. Cages are considered more hygienic than litter systems due to the separation of waste and animals [65,67]. However, all the birds were housed in the same space, so airborne bacteria and fungi could reach the cage hens too. Nevertheless, ingestion of litter and fecal material could have exposed the hens’ intestinal tracts to pathogens and initiated an immune response. In turn, the pen hens could have had increased fecal IgA concentrations to mediate an immune response against those intestinal pathogens. Further research should determine the impact of litter access on intestinal immune challenges and include a chronic stressor that is unrelated to housing conditions. Then, the impact of litter access and chronic stress can be separated and the use of secretory IgA as a biomarker for animal welfare confirmed. 

Even though housing conditions impacted the fecal IgA concentrations at 22 weeks of age, the plasma IgA concentrations did not differ. Secretory IgA may provide more insight in stress-induced immune system disruptions because IgA are most prominently active and present on mucosal surfaces including the intestinal tract [28]. IgA originating from the intestinal tract will be deposited in fecal matter as it passes through. Negative stressors, including heat stress [68,69,70,71] and stocking density [72,73,74,75] negatively impact the intestinal tract via damaged intestinal morphology and decreased microbiota diversity in commercial broilers and laying hens. These impacts on intestinal health could also impact the immune system, and, therefore, intestinal IgA via a reduction in IgA-secreting plasma cells (mature B-cells) or via direct interactions with intestinal glucocorticoids [26] indicating multiple possible mechanisms for the observed impacts of welfare status on IgA concentrations. Our results are the first to suggest an association between housing conditions and fecal IgA responses in laying hens, albeit only at 22 weeks of age. These results are a first step towards using secretory IgA as an indicator of welfare status in laying hens. Further research is needed to elucidate the impact of litter access and bird age on IgA concentrations.

Feather CORT assays have been used in several species including wild birds, broilers, and laying hens [20,21,22,23]. The feather CORT concentrations were higher in cage hens compared to the pen hens overall, however, feather CORT concentrations did not differ between the housing treatments at any individual timepoints. The latter is likely due to the low sample volume at individual timepoints. Our CORT concentrations indicate that layers from cages experienced more chronic stress than layers from pens. This difference possibly occurred due to repeated bouts of frustration or negative stress contributing to negative affective states. During these bouts of frustration or negative stress, the HPA axis releases glucocorticoids into the bloodstream, which are partially deposited into the feathers as they grow. The sum of these deposits is used to quantify stress over the whole feather growth period. Brown Nick laying hens that were housed at high stocking density, which is largely considered a chronic stressor in commercial poultry [75,76,77,78,79] had increased feather CORT at 10 weeks of age, when compared to birds that were housed at a lower stocking density [80]. In the current study, the stocking density differed considerably between the treatments, which could have contributed to the low feather CORT concentrations in pens compared to cage hens. While feather cover was not quantified as part of this experiment, birds that were housed in cages showed poorer feather coverage than birds that were housed in pen housing. It is possible that this poor feather coverage in cage birds would have made them more susceptible to cold stress during the cold period in the winter months (weeks 33–37) thereby increasing their feather CORT concentrations. Additional research should be done to determine if cold stress will impact feather CORT concentrations. Feather CORT concentrations also significantly decreased at week 29 of age compared to week 25 regardless of treatment. This was unexpected as feather CORT should increase continuously throughout life (as CORT keeps being deposited) unless the feather is regrown or replaced. It is possible that this observed decrease is methodologically related, however, all analyses were performed by the same researcher and the same protocol was followed. Feather CORT concentrations do rebound and reach a peak in week 43, which coincides with the cold spell that was observed from weeks 33 to 37 and likely reflects chronic stress that was caused during this period to birds in both housing systems. Overall, these results indicate that feather CORT shows promise as a viable physiological biomarker for chronic stress in laying hens. However, additional research is needed to confirm these results in commercial production systems and in other genetic strains. 

## 5. Conclusions

The birds’ responses in the current study suggest that conventional cages may induce negative affective states and emotions, reflected in somewhat increased fear and chronic stress responses, compared to enriched floor pens. However, anxiety was reduced in hens that were housed in conventional cages compared to the enriched pens. Simlar to previous research, these results highlight the importance of including a range of measures when assessing the impact of housing conditions on animal welfare. The novel measures of animal welfare that were tested in this study (Immunoglobulin A and feather corticosterone) indicate a downregulated immune response at 22 weeks of age (decreased fecal Immunoglobulin A) and increased chronic stress response (increased feather corticosterone) in conventionally caged hens compared to hens that were housed in enriched floor pens. However, fecal Immunoglobulin A concentrations require more research to confirm that the difference was not because of a mounted immune response due to exposure to litter. Overall, enriched pens resulted in improved laying hen immune responses at 22 weeks of age, decreased chronic stress, somewhat decreased fear, but increased anxiety indicating potential improvements in affective states (fear and chronic stress) in some aspects, but worsening in others (anxiety), and an improvement in the basic health and functioning (immune responses) when compared to conventionally-housed birds. Fecal (secretory) Immunoglobulin A and feather corticosterone quantification show some contrasting responses in line with expectations and behavioral outcomes, yet need further confirmation before application as a routine measure for emotion and affective state in laying hens. 

## Figures and Tables

**Table 1:** Method that was used during attention bias (ABT) testing. The birds were tested in groups of three and the testing procedure differed depending on the number of birds who began feeding following the first alarm call.

Scenario	Procedure	Total Test Duration	Data Recorded
Testing begins	Play first alarm call	300 s	n/a
No. birds begin feeding	Allow test to run 300 s	300 s	All birds receive 300 s maximum latency to begin feeding
One bird begins feeding	Play first alarm call and allow test to run for 300 s	300 s	Latency to begin feeding for bird that began feeding. Other two birds receive maximum latency of 300 s
Two birds begin feeding	Play first alarm call and allow test to run for 300 s. Play second alarm call at 300 s and allow test to run until 420 s.	420 s	Latencies to begin feeding for two birds. Third bird receives maximum latency of 300 s. Latencies to resume feeding for two birds that began feeding if they feed before 420 s.
All three birds begin feeding before 270 s	Play first alarm call and allow test to run until the third bird begins feeding. Allow birds to feed for 5 s and play second alarm call. Allow test to run until 300 s	300 s	Latencies to begin feeding. Latencies to resume feeding for all three birds if they resume feeding prior to 300 s.
All three birds begin feeding between 270–300 s	Play first alarm call and allow test to run until the third bird eats. Allow birds to feed for 5 s and play second alarm call. Extend testing duration to 420 s.	420 s	Latencies to begin feeding. Latencies to resume feeding for all three birds if they resume feeding before 420 s.

n/a: not applicable.

**Table 2:** Responses in the attention bias test at 30 weeks of age for laying hens that were housed in conventional cages (*n* = 36) or enriched floor pens (*n* = 36).

Measure	Conventional Cage	Enriched Floor Pen
Latency to begin feeding (s)	99.17 ± 19.23 ^a^	145.97 ± 24.52 ^b^
Latency to resume feeding (s)	54.21 ± 13.63	54.13 ± 13.87
Birds begin feeding (%)	91.66 ^A^	77.77 ^B^
Birds resume feeding (%)	87.87 ^a^	82.14 ^b^
Vigilance behavior score (0–4 score) ^1^	2.35 ± 0.26	2.33 ± 0.27
Freeze (% birds)	55	35
Erect (% birds)	55	44
Neck stretch (% birds)	65	55
Look (% birds)	80	72

^1^ Birds were scored either 0 (not observed) or 1 (observed) for each of four vigilance behavior characteristics (erect posture, neck stretching, freezing, and looking around), resulting in a vigilance score between 0 (no vigilance behavior observed) and 4 (all vigilance behaviors observed). The data are displayed as raw means ± SEM. ^a, b^ Row values lacking a common superscript differ at *p* < 0.05. ^A, B^ Row values lacking a common superscript tend to differ at *p* < 0.10.

## Data Availability

Data are available from the corresponding author upon reasonable request.
